# A Plant-Based Diet and Cardiovascular Disease, Coronary Heart Disease, and Stroke: A Systematic Review and Meta-Analysis of Prospective Cohort Studies

**DOI:** 10.3390/nu18172779

**Published:** 2026-08-25

**Authors:** Youngyo Kim, Yoona Kim

**Affiliations:** Department of Food and Nutrition, Institute of Agriculture and Life Science, Gyeongsang National University, Jinju 52828, Republic of Korea; youngyokim@gnu.ac.kr

**Keywords:** plant-based diets, cardiovascular disease, coronary heart disease, stroke, meta-analysis

## Abstract

**Background/Objectives**: This study aimed to examine the association between a plant-based diet (PBD) and the incidence of total cardiovascular disease (CVD), coronary heart disease (CHD), and stroke in a meta-analysis of prospective cohort studies. **Methods**: We searched the PubMed, ISI Web of Science and Scopus databases up to February 2026 to identify prospective cohort studies assessing the association between a PBD and the incidence of total CVD, CHD, and stroke. **Results**: A total of twenty-two articles (nineteen cohort studies), including 846,589 subjects and 139,652 cases, were included in the meta-analysis. People with the highest adherence to overall PBD had a 10% lower risk of CVD than those with the lowest adherence (relative risk [RR] = 0.90, 95% CI: 0.85–0.95). Adherence to a healthful plant-based diet (hPBD) was inversely associated with CVD risk (RR = 0.83, 95% CI: 0.76–0.90), whereas adherence to an unhealthful plant-based diet (uPBD) was positively associated with CVD risk (RR = 1.13, 95% CI: 1.03–1.24). High compliance with overall and a healthful PBD was associated with a 9% (RR = 0.91, 95% CI: 0.85–0.96) and 23% lower risk (RR = 0.77, 95% CI: 0.72–0.84) of CHD development compared with low compliance, respectively. High compliance with a uPBD was associated with a 24% higher risk of CHD than low compliance. We found no significant association with stroke risk. **Conclusions**: This meta-analysis indicated that a PBD was inversely associated with the risk of CVD and CHD. A hPBD showed an inverse association, whereas a uPBD showed a positive association between cardiovascular health. Further well-designed interventions are needed to clarify these associations.

## 1. Introduction

Cardiovascular disease (CVD) is a leading cause of global morbidity and mortality. CVD global mortality elevated from 13.1 million to 19.2 million between 1990 and 2023. It is estimated to reach approximately 35.6 million annually by 2050 [1]. CVD is primarily attributable to coronary heart disease (CHD) and stroke. CHD and stroke account for 85% CVD mortality [2,3]. CVD imposes an enormous economic burden on health care systems [4].

CVD risk factors include non-modifiable factors (e.g., age, genes, and gender) and modifiable factors (e.g., smoking, excessive alcohol intake, unhealthful diet, physical inactivity, high blood pressure, high blood cholesterol, obesity, and diabetes) [5]. Dietary modification can play a beneficial role in CVD prevention, reducing a substantial economic burden on health care systems. A plant-based diet (PBD) has drawn attention as a CVD prevention strategy in recent years. A PBD consists of plant-based foods such as whole grains, vegetables, fruits, legumes, seeds, and nuts. In particular, a healthful plant-based diet (hPBD) includes whole grains, fruits, vegetables, nuts, legumes, vegetable oils, and tea/coffee. An unhealthful plant-based diet (uPBD) includes fruit juices, sugar-sweetened beverages, refined grains, potatoes, and sweets/desserts [6].

A higher plant-based diet index (PDI) score was associated with a lower risk of CVD events during a mean follow-up of 25 years, in the Atherosclerosis Risk in Communities (ARIC) study of 12,168 subjects [7]. Moreover, a hPBD was associated with a reduced risk of CVD during a mean follow-up of 12.3 years, in an analysis of 158,408 subjects aged 40 to 69 years from the UK Biobank prospective cohort study [8]. Meanwhile, a higher intake of unhealthful and ultra-processed plant-based foods was associated with an increased incidence of CHD during the median follow-up period of 9 years in the French NutriNet-Santé cohort study of 63,831 subjects aged over 35 years [9]. However, the EPIC-Oxford study over 18 years of follow-up showed the association between a vegetarian diet and increased risk of stroke in comparison with meat eaters, when analyzing 16, 254 subjects aged over 39 years [10].

Given the conflicting findings on the association between a PBD and the incidence of total CVD, CHD, and stroke in prospective cohort studies, this study aimed to conduct a comprehensive meta-analysis to examine this association.

## 2. Materials and Methods

### 2.1. Literature Search Strategy

To identify the studies suitable for this meta-analysis, the PubMed, ISI Web of Science and Scopus databases were searched until February 2026. The search terms were “(plant-based diet OR vegetarian OR vegan) combined with (cardiovascular disease OR coronary heart disease OR coronary artery disease OR stroke).” (Supplementary Table S1). We additionally reviewed the reference lists of the retrieved articles for further eligible studies. The current meta-analysis was registered at PROSPERO (CRD420261302639).

### 2.2. Selection of Study

Studies were considered eligible for inclusion in this meta-analysis based on the following criteria: prospective cohort design; a PBD as exposure; incidence of CVD, CHD, and stroke as outcomes; relative risks (RRs); and 95% confidence intervals (CIs) (Supplementary Table S2). When duplicate cohort studies were present, we included the study with the greater number of participants in the analysis.

### 2.3. Data Extraction

All authors independently extracted data from original articles according to the Preferred Reporting Items for Systematic reviews and Meta-Analyses (PRISMA) statement [11]. The extracted information includes: the first author’s last name, the country or region where the study was conducted, the age of subjects, the study periods or follow-up years, the sex of subjects, the number of subjects, the number of cases, the diet classification category, estimates and their 95% CIs, and adjusted variables.

### 2.4. Quality Assessment

All authors independently assessed study quality using a Newcastle–Ottawa Scale [12]. Points were awarded for three items: participant selection (4 points), comparability between cohorts (2 points), and disease identification (3 points), with a maximum score of 9 points. Studies that received a score of 7 or higher were considered high-quality studies. All authors independently evaluated the quality, and any discrepancies were resolved through a discussion and review of the original paper.

### 2.5. Statistical Analysis

Pooled relative risk and its 95% CI were calculated for the highest *versus* the lowest adherence to a PBD using the DerSimonian and Laird random effects models [13]. Heterogeneity among the studies was measured using Cochran’s *Q* statistic [14], and the *I*^2^ statistic [15] was used to quantify the degree of heterogeneity. To assess whether the influence of a specific study was not overly significant, we conducted a sensitivity analysis by integrating the results of the remaining studies while excluding each study in turn. Where possible, we performed subgroup analyses based on sex, region or follow-up years. The results of studies comparing vegetarians and meat consumers (not comparing the highest *versus* the lowest PBD pattern scores) were collected separately and meta-analyzed. Publication bias was verified by Begg’s [16] and Egger’s regression asymmetry test [17]. All statistical analyses were conducted using STATA 17.0 software, and a *p*-value of less than 0.05 was considered an indicator of significance.

## 3. Results

### 3.1. Study Characteristics

**Figure 1:**
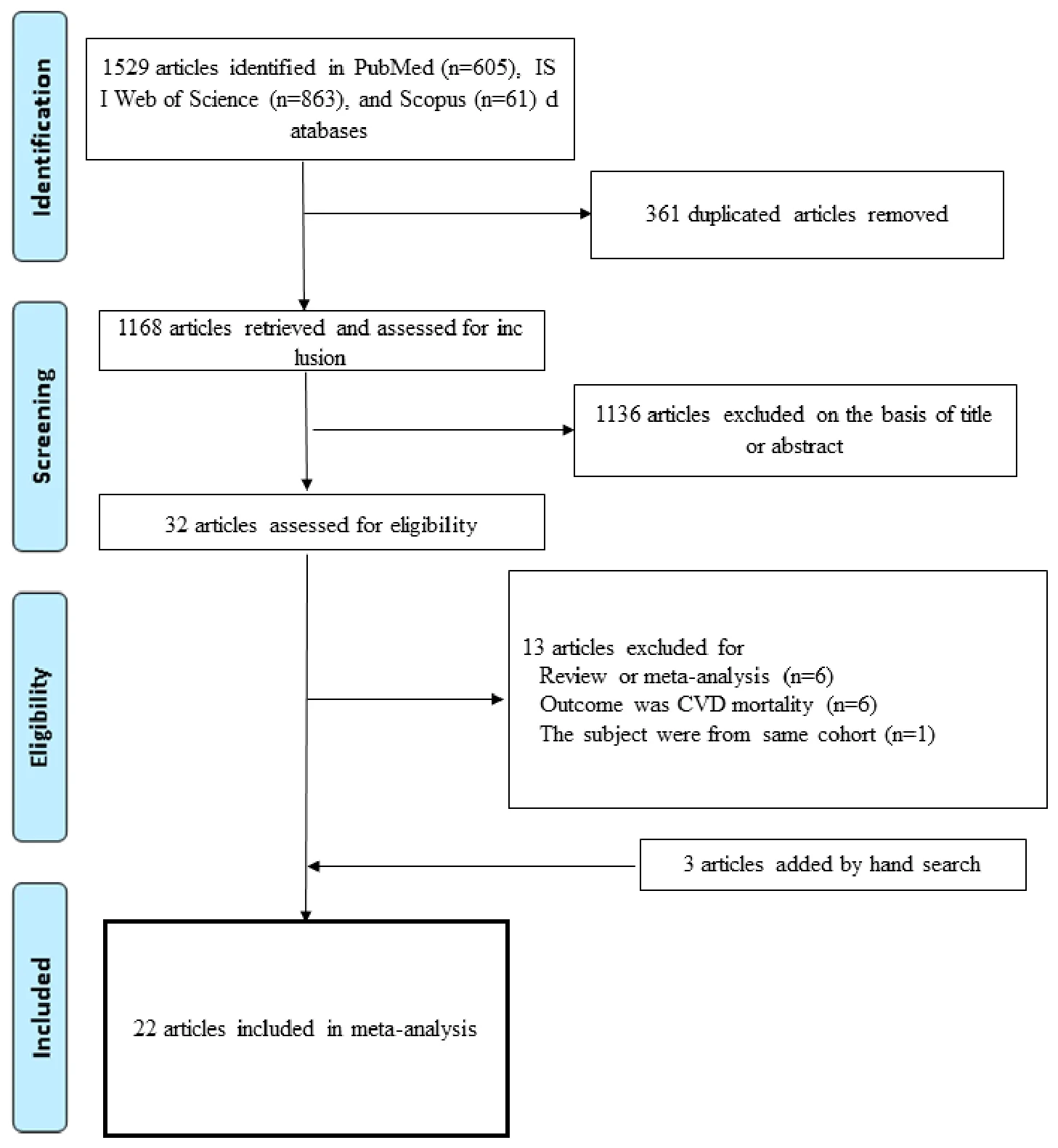
The flow chart of study selection procedure.

The study’s search and selection process was shown in Figure 1. We included twenty-two articles in the meta-analysis [7,9,10,18,19,20,21,22,23,24,25,26,27,28,29,30,31,32,33,34,35,36]. Table 1 presented the basic characteristics of prospective cohort studies examining the association between a PBD and CVD incidence. Eight articles reported results for a PBD, a hPBD, and a uPBD [7,21,24,28,29,31,33,34]; two reported results for a hPBD and a uPBD together [9,32]; and the rest reported results for only one of a PBD, a hPBD, and a uPBD [10,18,19,20,22,23,25,26,27,30,35,36]. Looking at the regions where the research were conducted, 14 studies were in the United States [7,9,19,20,21,22,23,24,25,26,27,29,31,32,35], five in Europe [9,10,18,28,30], and three in Asia [33,34,36]. In most cases, results were reported as comparisons of adherence to a PBD, and four articles reported comparisons between vegetarian and non-vegetarian diets [10,18,26,36]. The subjects were 18 years or older. The follow-up period ranged from 2 to 32 years with a median of 11.4 years. The Newcastle–Ottawa Scale scores ranged from 6 to 9, indicating a mean score of 7.7 (Supplementary Table S3). All studies reported results adjusted for age and smoking; most studies controlled for physical activity (n = 21) or alcohol consumption (n = 20), and many also adjusted for body mass index (BMI; n = 19) and energy intake (n = 15).

### 3.2. PBD and Risk of CVD

**Figure 2:**
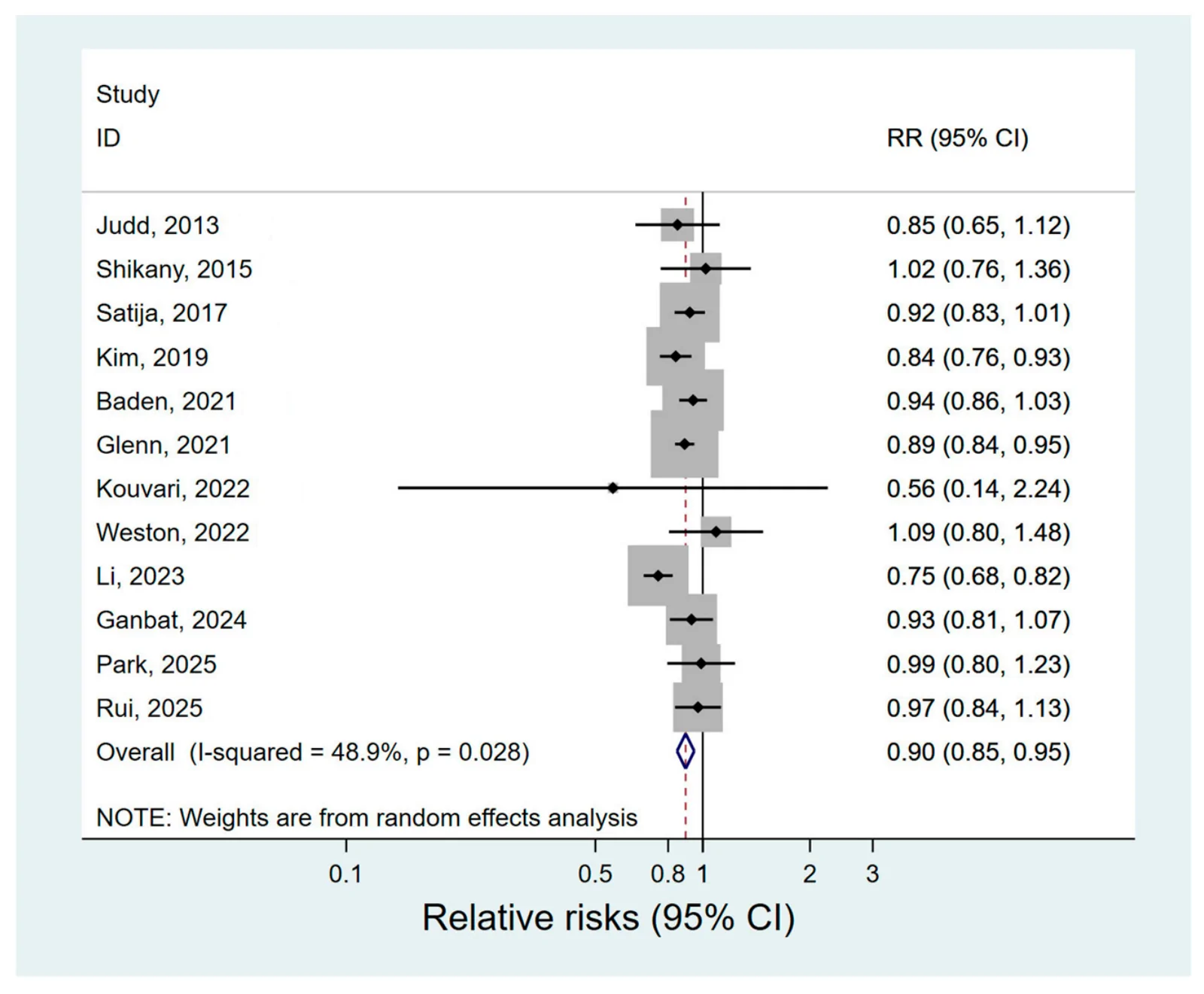
Forest plots of the prospective cohort studies of cardiovascular disease for high *versus* low adherence to a plant-based diet (PBD) [7,19,20,21,24,25,28,29,31,33,34,35].

Twelve articles analyzing the association between a PBD and CVD incidence were included in the meta-analysis [7,19,20,21,24,25,28,29,31,33,34,35]. When comparing the highest and lowest PBD scores, the pooled RR was 0.90 (95% CI: 0.85–0.95), indicating significant heterogeneity (*I*^2^ = 48.9%, *p* = 0.03) (Figure 2). The observed significant heterogeneity disappeared (*I*^2^ = 0.0%, *p* = 0.69) when the study by Li et al. (2023) [31], which showed a strong inverse association, was excluded, and the pooled RR remained largely unchanged at 0.91 (95% CI: 0.88–0.94). Subgroup analysis by sex revealed a significant inverse association only in women (RR = 0.90, 95% CI: 0.85–0.94), but the sex difference was not statistically significant (*p* for difference = 0.38) (Table 2). When analyzed by the region where the study was conducted, significant results were found only in the U.S. (RR = 0.88, 95% CI: 0.82–0.94), but differences based on region were also not statistically significant (*p* for difference > 0.2 in all comparisons) (Table 2). There was no publication bias for the association between a PBD and CVD incidence (Begg’s *p* = 0.73, Egger’s *p* = 0.48).

### 3.3. hPBD and Risk of CVD

**Figure 3:**
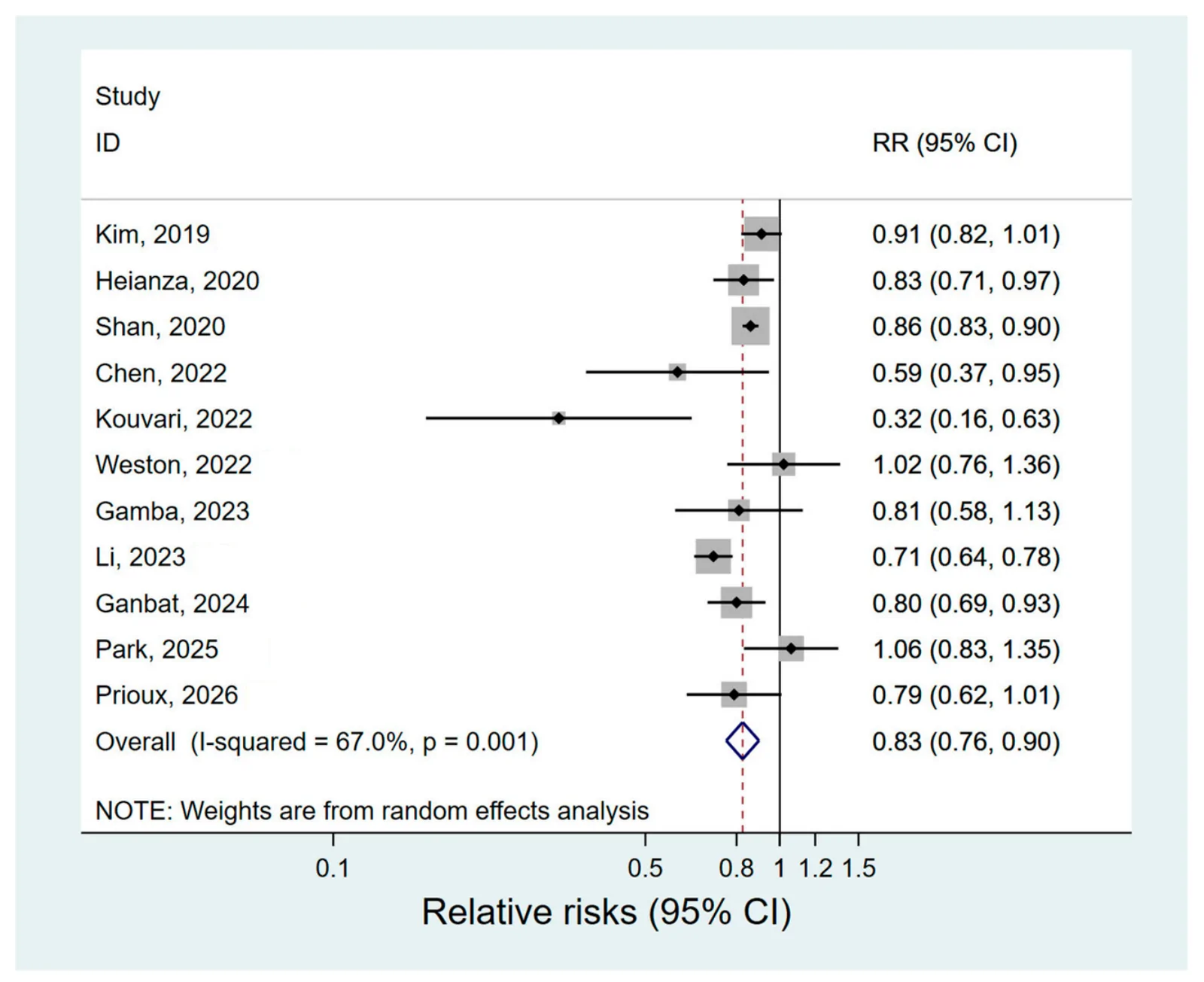
Forest plots of the prospective cohort studies of cardiovascular disease for high *versus* low adherence to a healthful plant-based diet (hPBD) [7,9,22,23,27,28,29,30,31,33,34].

Eleven articles providing the association between hPBD and CVD incidence were included in the meta-analysis [7,9,22,23,27,28,29,30,31,33,34]. The pooled RR for the highest *versus* lowest adherence to hPBD was 0.83 (95% CI: 0.76–0.90), with significant heterogeneity (*I*^2^ = 67.0%, *p* < 0.01) (Figure 3). After excluding two studies [28,31], significant heterogeneity disappeared (*I*^2^ = 15.0%, *p* = 0.31), and the pooled RR was 0.86 (95% CI: 0.82–0.91). In the subgroup analysis by region, significant inverse associations were found in both the U.S. (RR = 0.83, 95% CI: 0.74–0.93) and Europe (RR = 0.75, 95% CI: 0.60–0.94) (Table 2). In contrast, in Asia, the results were not significant (RR = 0.91, 95% CI: 0.69–1.19). However, the difference was not statistically significant (*p* for difference >0.5 in all comparisons) (Table 2). No publication bias was observed in Begg’s (*p* = 0.28) and Egger’s (*p* = 0.37) test.

### 3.4. uPBD and Risk of CVD

**Figure 4:**
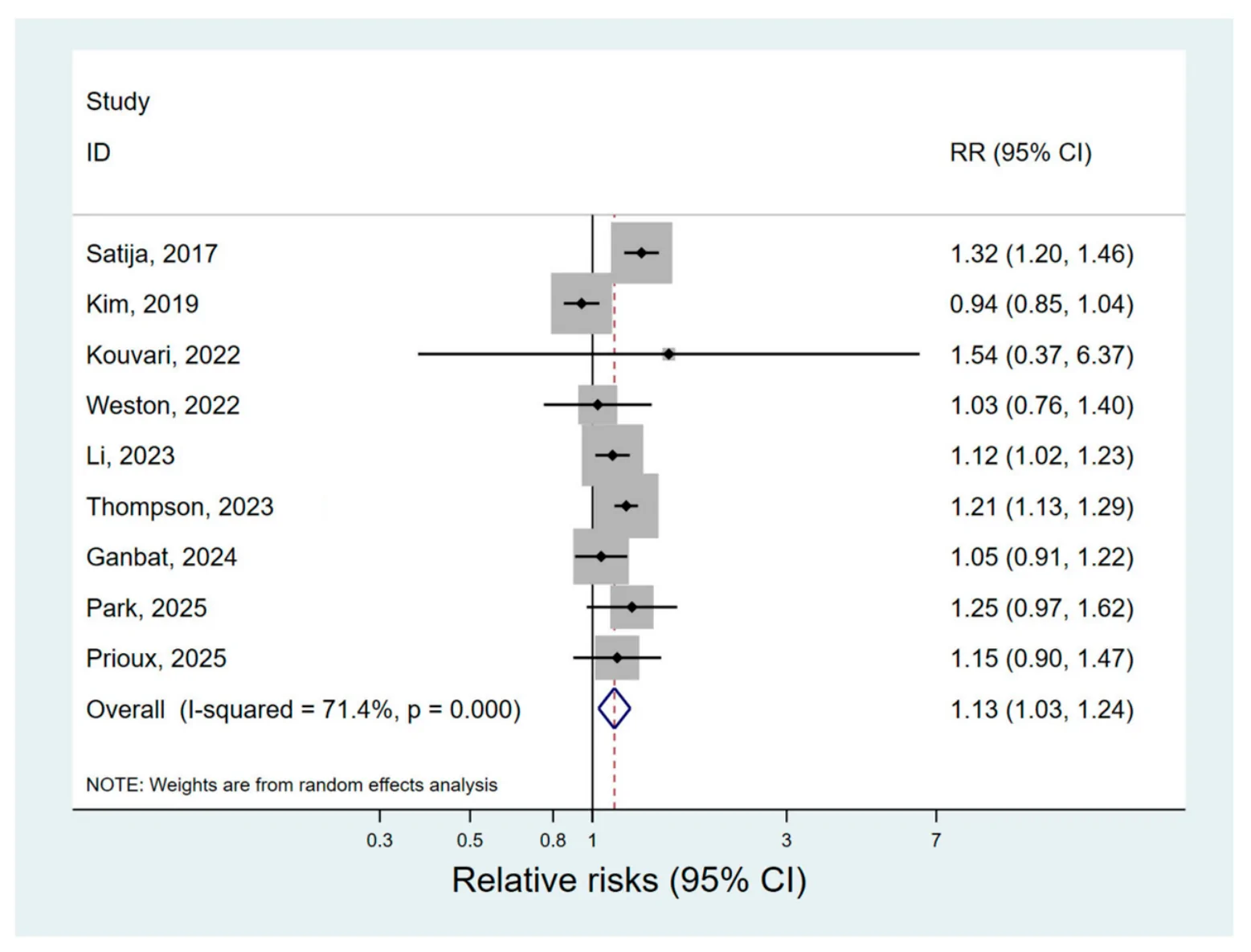
Forest plots of the prospective cohort studies of cardiovascular disease for high *versus* low adherence to an unhealthful plant-based diet (uPBD) [7,9,21,28,29,31,32,33,34].

Eleven articles investigating the association between a uPBD and CVD incidence were included in the meta-analysis [7,9,21,28,29,31,32,33,34]. The pooled RR for the highest and lowest uPBD scores was 1.13 (95% CI: 1.03–1.24), showing significant heterogeneity (*I*^2^ = 71.4%, *p* < 0.01) (Figure 4). When the only study showing an RR value less than 1 was excluded [7], significant heterogeneity disappeared (*I*^2^ = 29.7%, *p* = 0.19), and the pooled RR showed a slightly stronger positive association (RR = 1.18, 95% CI: 1.11–1.26). In the subgroup analysis by sex, a significant positive association was observed only in women (RR = 1.53, 95% CI: 1.31–1.79), but the difference was not statistically significant (*p* for difference = 0.11) (Table 2). We found no difference by geographical region (*p* for difference >0.5 in all comparisons). There was no indication of publication bias (Begg’s *p* = 0.92, Egger’s *p* = 0.77).

### 3.5. Risk of CHD and Stroke

**Figure 5:**
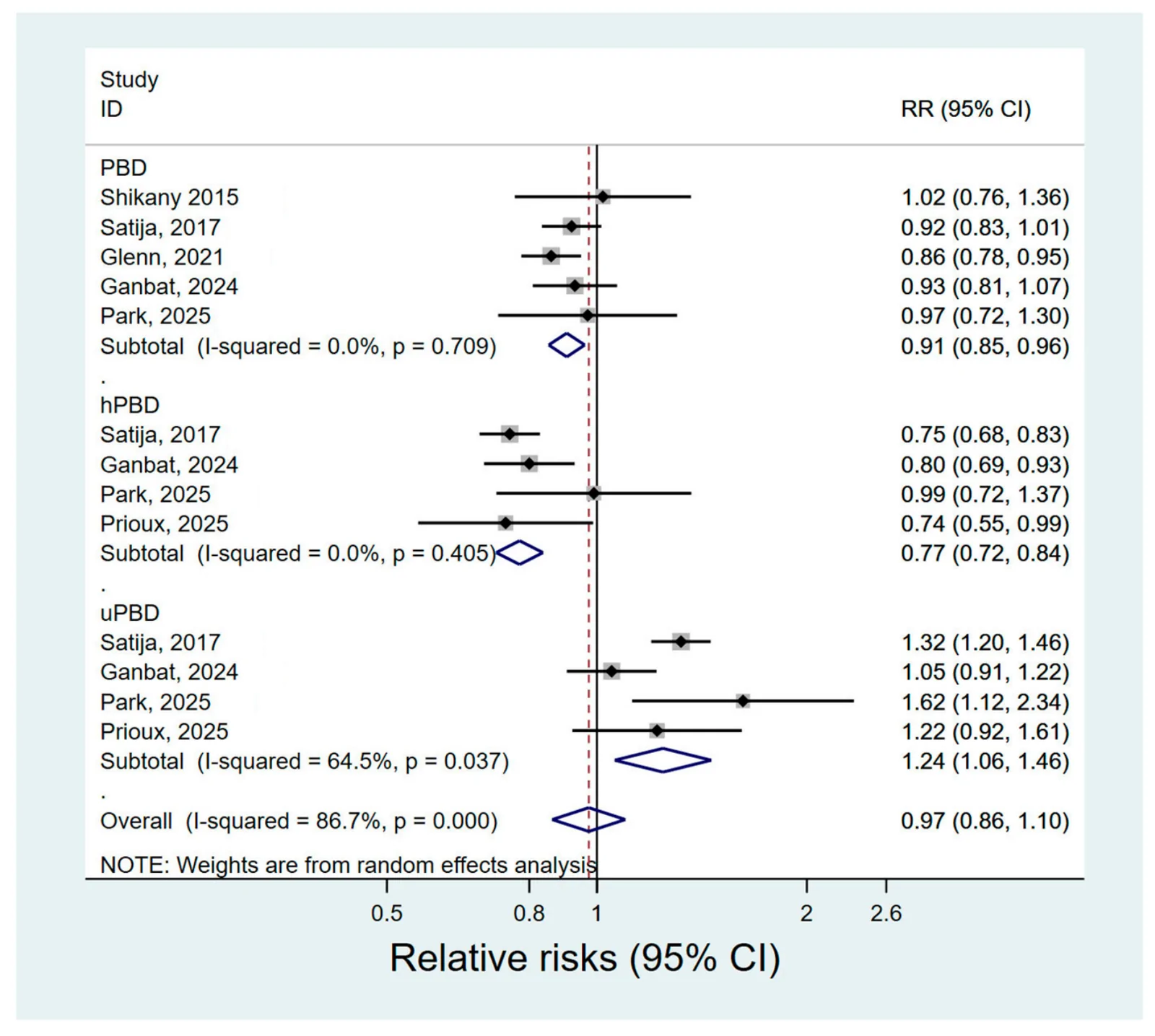
Forest plots of the prospective cohort studies of coronary heart disease for high *versus* low adherence to a plant-based diet (PBD), a healthful PBD (hPBD), and an unhealthful PBD (uPBD) [9,20,21,25,33,34].

Six articles reported the RRs of CHD incidence for a PBD, a hPBD, or a uPBD [9,20,21,25,33,34]. When comparing the group with the highest dietary adherence to the group with the lowest, a PBD (RR = 0.91, 95% CI: 0.85–0.96) and a hPBD (RR = 0.77, 95% CI: 0.72–0.84) showed inverse associations between diet and CHD risk, and there was no significant heterogeneity (Figure 5), whereas a uPBD showed a positive association (RR = 1.24, 95% CI: 1.06–1.46), with significant heterogeneity (*I*^2^ = 64.5%, *p* = 0.04) (Figure 5).

**Figure 6:**
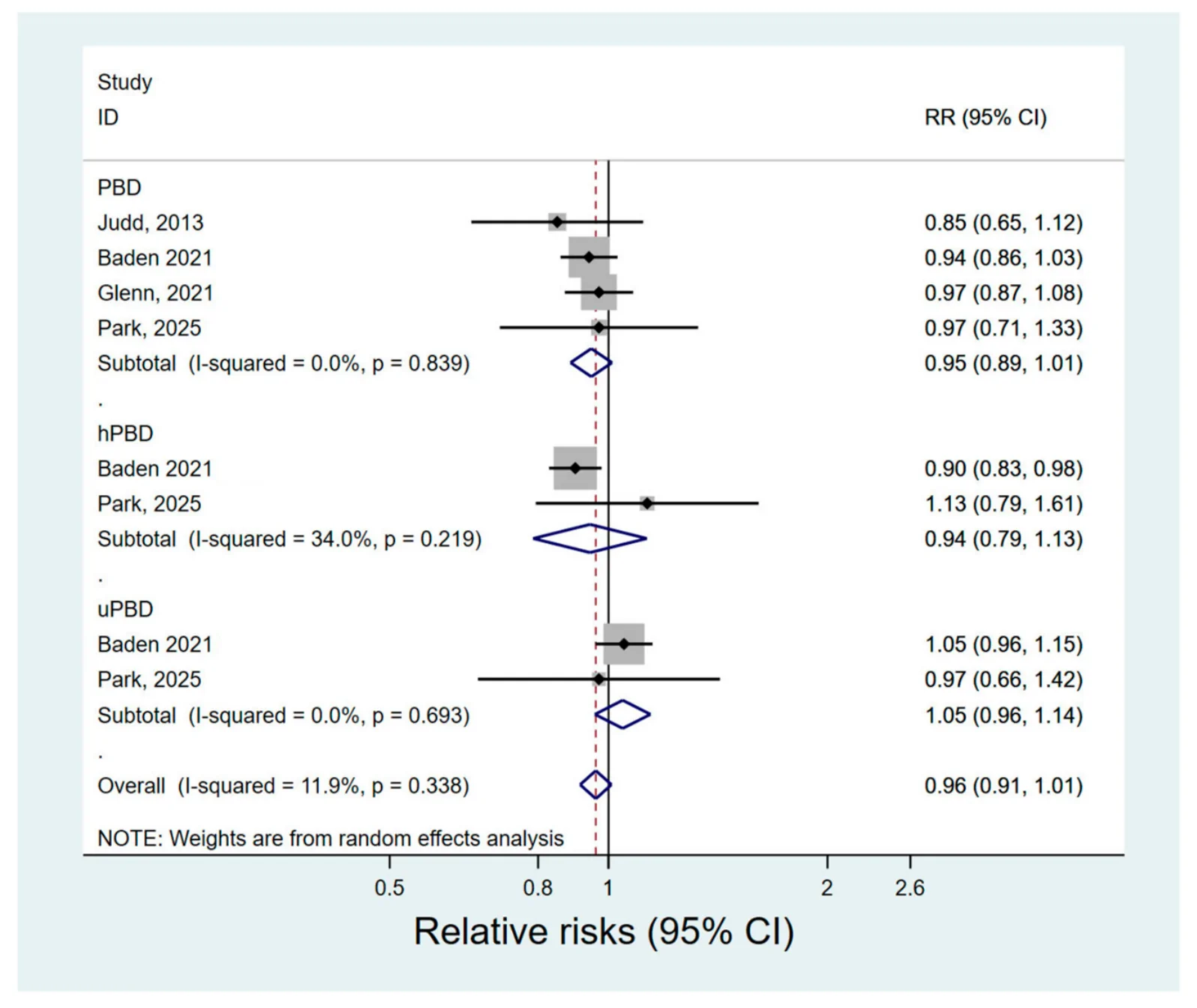
Forest plots of the prospective cohort studies of stroke for high *versus* low adherence to a plant-based diet (PBD), a healthful PBD (hPBD), and an unhealthful PBD (uPBD) [19,24,25,34].

Four articles provided the RRs of stroke incidence for a PBD, a hPBD, or a uPBD [19,24,25,34], and the forest plot was shown in Figure 6. We did not observe a significant association with stroke risk in a PBD (RR = 0.95, 95% CI: 0.89–1.01), a hPBD (RR = 0.94, 95% CI: 0.79–1.13), and a uPBD (RR = 1.05, 95% CI: 0.96–1.14).

### 3.6. Vegetarians Versus Meat-Eaters

**Figure 7:**
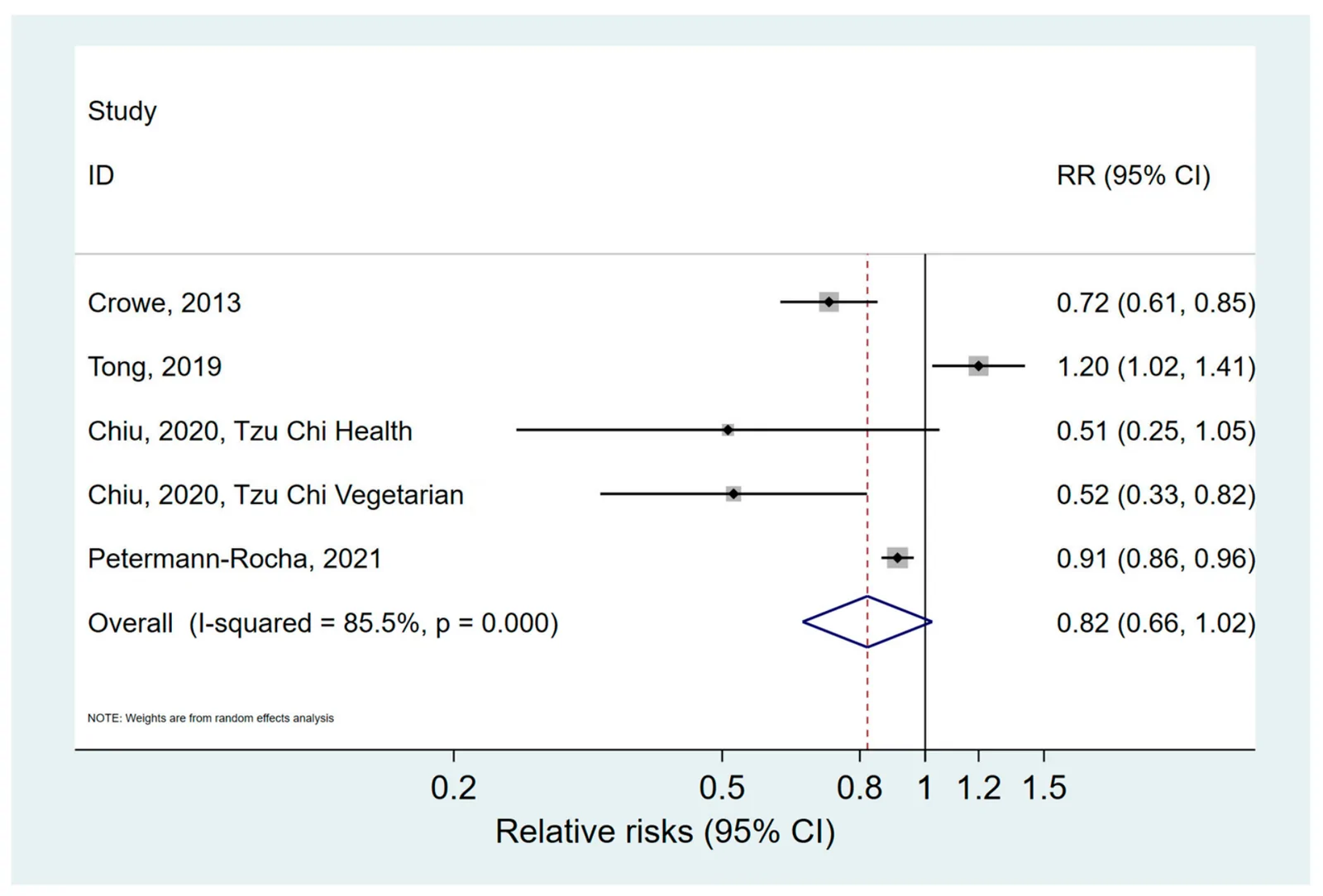
Forest plots of the prospective cohort studies of cardiovascular disease for vegetarians compared with meat eaters [10,18,26,36].

Four articles reporting RRs for CVD comparing meat eaters and vegetarians were included in the meta-analysis [10,18,26,36]. The pooled RR was 0.82 (95% CI: 0.66–1.02) with significant heterogeneity (*I*^2^ = 85.5%, *p* < 0.01) (Figure 7). There was no publication bias (Begg’s *p* = 0.81, Egger’s *p* = 0.50).

## 4. Discussion

The present meta-analysis examined the association between a PBD and the incidence of total CVD, CHD, and stroke, including 19 prospective cohort studies with 846,589 subjects. We observed that overall, a PBD was associated with reductions of 10% and 9% in the risks of CVD and CHD, respectively. A hPBD was associated with a 17% and 23% reduction in CVD and CHD risk, respectively.

The inverse association between a PBD and the incidence of CVD and CHD can be linked to the protection against CVD. This diet can reduce CVD risk factors by lowering blood pressure [37], lipids [38,39,40], inflammation [41], glucose [42], body weight [42], and trimethylamine-N-oxide (TMAO) [43], and by increasing gut microbiota diversity [41] and endothelial function [44,45,46]. The recent umbrella review by Capodici et al. (2024) [47] showed that vegetarian and vegan diets were associated with improved glucose, lipid, body weight/BMI, inflammatory markers, and a lower risk of ischemic heart disease [47].

High blood pressure could affect CVDs and CHD risk [48]. A meta-analysis of 15 randomized clinical trials (RCTs) involving 856 subjects found that a PBD could play a role in blood pressure management. A vegetarian diet decreased the systolic blood pressure (SBP) and diastolic blood pressure (DBP) by 2.66 mmHg and 1.69 mmHg, respectively, compared with an omnivorous diet [37].

A PBD has been shown to lower blood lipid levels in human studies [38,39,40]. In a RCT of subjects aged an average of 67.3 years with coronary artery disease (CAD) treated with percutaneous coronary intervention (PCI) and on optimal medical therapy, a lacto-ovo-vegetarian diet for 4 weeks decreased plasma lipotoxic lipids (triacylglycerols, glycerophospholipids, cholesteryl ester, and ceramide) and increased triacylglycerols with long-chain polyunsaturated fatty acyls compared with an isocaloric meat diet [38]. A PBD contains phytochemicals. In a systematic review of RCTs examining 3478 hypertriglycemia subjects aged 18–70 years, phytochemicals supplementation (e.g., curcumin, berberine, resveratrol, catechins, epicatechin, anthocyanin, quercetin, bergamot, soy isoflavones, and so on) improved lipoid profiles by decreasing serum total cholesterol (TC), low-density lipoprotein cholesterol (LDL-C), and increasing serum high-density lipoprotein cholesterol (HDL-C) [39]. A PBD rich in phytosterols consists of plant sterols and stanols, which are a class of phytochemicals. In a meta-analysis of 124 RCTs, the average phytosterols intake of 0.6–3.3 g per day gradually decreased serum LDL-C levels by approximately 6–12% [40]. However, adherence to a PBD typically provides about 300–600 mg of phytosterol per day [49].

A PBD is high in mono- and poly-unsaturated fatty acids and low in saturated fats. The main sources of fats in a PBD are plant-based oils, nuts, and seeds. Olive oil, as a plant-based oil, is high in monounsaturated fatty acids, containing 70% to 73% of its total fat. A meta-analysis of 8 RCTs showed that olive intake improved endothelial function by increasing flow-mediated dilation compared with controls [45]. Nuts are rich in mono- and poly-unsaturated fatty acids, but they are low in saturated fats. A meta-analysis of 10 RCTs showed that nut consumption improved endothelial function [46].

A hPBD abundant in dietary fiber, comprising whole grains, vegetables, fruits, legumes, seeds, and nuts, could enhance gut microbiota diversity, reduce inflammation, and improve glucose control. In the Chinese prospective study, the positive association between short-term hPDI and microbial alpha diversity was observed. The short-term hPDI was derived from 3-day 24 h dietary recalls. Higher microbial alpha diversity was associated with lower CVD risk biomarkers, including fasting insulin, HDL-C, LDL-C, TG (triglycerides), and hs-CRP (high-sensitivity C-reactive protein) during the 3-year follow-up period [41]. Moreover, a meta-analysis of 20 RCTs involving 1878 people aged 28–64 years with CVDs showed that a vegetarian diet for an average of 6 months reduced body weight, hemoglobin A1c, and LDL-C during the intervention periods ranging from 2 to 24 months [42].

Choline, lecithin, and L-carnitine, which are rich in red meat, eggs, fish, and dairy products are broken down into trimethylamine (TMA) by gut microbiota. TMA is converted to TMAO by flavin monooxygenases in the liver. TMAO is known to increase CVD risk [50]. A recent randomized, crossover intervention in subjects with an average age of 51 years showed that a 6-day PBD significantly attenuated serum TMAO levels compared with a red meat diet [43]. Meanwhile, we observed no significant RR for CVD risk comparing meat eaters and vegetarians with significant heterogeneity. Red meat is rich in high bioavailability protein, iron, zinc, and vitamin B12, which can be limited or absent in a PBD [51]. Recent RCTs have shown that unprocessed beef or lean beef added to healthy dietary patterns benefited body weight maintenance [52], gut microbiota diversity, and TMAO reduction [53]. The present study found that a uPDI was associated with increased CVD and CHD risk. In line with our findings, the Framingham Offspring Cohort Study of 3121 subjects with a baseline age of 55 years showed that the highest intake of refined grains was associated with increased waist circumference compared with the lowest intake during the mean follow-up period of 13 years [54]. Consistently, a meta-analysis of 25 RCTs during the intervention periods ranging from 2 to 16 weeks showed that replacing refined grains with whole grains decreased LDL-C, TC, TG, hemoglobin A1c, and CRP [55].

Furthermore, several meta-analyses of prospective studies or RCTs have indicated the association between carbohydrate quality and CVD risk. In general, refined grains have a higher glycemic index than whole grains [56]. High glycemic index (GI)/ glycemic load (GL) was associated with an increased CVD risk [57,58]. In a meta-analysis of 27 RCTs with an average intervention period of 12 weeks, 1617 subjects with type 1 or 2 diabetes who adhered to low-GI/GL dietary patterns showed decreased body weight, hemoglobin A1c, fasting glucose, LDL-C, TG, SBP, and CRP [58].

The interpretation of the present findings can be supported by emerging cardiovascular biomarkers and multidimensional cardiovascular risk assessment approaches. Kwiatkowska et al. (2023) [59] estimated CVD risk using newly developed metrics based on assessments of biochemical parameters in healthy subjects aged 18–39 years who adhered to one of 4 dietary patterns: vegans, lacto- or ovo-vegetarians, pescatarians, and omnivores. The vegan group was likely to have the lowest CVD risk compared with other diet groups [59]. Very recently, Kim et al. (2025) [60] examined the association between a PBD and CVD risk in the ARIC study and Framingham Heart Study Offspring cohort using a proteomics approach to investigate the underlying mechanism. Thrombospondin-2 (THBS2) and N-terminal pro-BNP(NPPB) were negatively associated with a hPBD. Moreover, THBS2 and N-terminal pro-BNP were positively associated with CVD risk [60].

The present study observed no significant association between a PBD and stroke risk. High blood pressure is a major contributor to stroke risk [61,62]. PBDs included in the present meta-analysis might not be strong enough to lower blood pressure, which might lead to limited stroke risk reduction.

The population-based Rotterdam Study indicated that a hPBD combined with positive lifestyle modifications (e.g., smoking cessation, adequate physical activity, moderate sleep duration) is vital for personalized CVD prevention [63]. Wang et al. (2026) [63] examined the association between the healthy plant-based diet-lifestyle score and CHD risk in the population-based Rotterdam Study. They observed the association between a higher healthy plant-based diet-lifestyle score and lower CHD risk during a mean follow-up of 15.2 years among 7764 subjects [63].

This study found an inverse association between a hPBD and CVD, whereas a uPBD was positively associated with CVD. When comparing food composition and scoring systems, the uPBD’s higher sugar content than the hPBD may have influenced the differing associations with the risk of CVD development. In addition, studies have shown improvements in CVD measures with a low-carbohydrate diet, independent of protein source. This may explain why a hPBD is associated with CVD [64,65,66,67].

The present study has strengths. We comprehensively assessed the association between a PBD and risks for CVD, CHD, and stroke in a prospective cohort design with carefully controlled potential confounders and lower recall or selection bias than in other observational study designs. We carefully attempted to include the most recent large prospective cohort studies with a substantial number of subjects, indicating that the present meta-analysis showed significant statistical power. We ensured that no duplicate cohorts are included in meta-analyses of the same dietary pattern. We also refrained from including multiple values from the same cohort in meta-analyses. Furthermore, the random-effects model [68] was used to account for and manage heterogeneity across the prospective cohort studies.

However, this study also has limitations. The prospective cohort studies in the meta-analyses used self-reported dietary data, which might not reflect changes in long-term dietary behaviors; thus, potential measurement errors and reporting bias cannot be ruled out. Secondly, although most studies included in the meta-analysis reported results adjusted for confounding factors such as age, smoking, physical activity, alcohol consumption, BMI, and energy intake, it is still possible that residual confounding factors exist. Thirdly, although most studies included in the meta-analysis calculated PBD, hPBD, and uPBD scores using the food classification and scoring system proposed by Satija et al. [6], the types of foods included in each food group may vary across cultures in which the studies were conducted. Therefore, caution should be exercised when interpreting the results, keeping in mind that misclassification arising from this may have influenced the integrated estimates. Finally, regarding the analysis of CHD and stroke, caution is warranted when interpreting the results, as the number of included studies was limited.

## 5. Conclusions

In conclusion, this meta-analysis provides the inverse associations between a PBD and the incidence of CVD and CHD. Specifically, high adherence to a hPBD was inversely associated with CVD risk. High adherence to a uPBD was associated with CVD risk. These findings indicate how important diet quality is for distinguishing healthful from less healthful food choices within a PBD when assessing their associations with CVD risk. However, potential residual confounding from overall dietary quality and lifestyle factors should be acknowledged when interpreting these findings. In addition, it should be noted that a uPBD has a higher sugar content than a hPBD, which may have influenced the results. Future well-designed interventions targeting a hPBD and a uPBD should be conducted to clarify the prevention of CVD.

## Figures and Tables

**Table 1:** Characteristics of prospective cohort studies included in the meta-analysis of a plant-based diet (PBD) and incidence of cardiovascular disease (CVD).

First Author, Year	Country	Cohort Name	Follow-Up Duration (Years)	Age Range (Years)	Sex	Study Size		Outcome	Diet Category	Adjustment for Covariates
						Subjects	No. of Cases			
Crowe, 2013 [18]	Europe	EPIC–Oxford study	11.6	≥20	Men and women	44,561	1235	IHD	Vegetarian compared with non-vegetarians	Age, sex, method of recruitment, and region of residence, smoking, alcohol, physical activity, educational level, Townsend Deprivation Index and the use of oral contraceptives or hormone therapy for menopause (only in women), BMI
Judd, 2013 [19]	US	REGARDS cohort study	5.7	≥45	Men and women	20,251	490	Stroke	Plant-based pattern	Age, race, region, sex, and age–race interaction, income and education, total energy, smoking and sedentary behavior (watching >4 h of television per day)
Shikany, 2015 [20]	US	REGARDS cohort study	5.8	≥45	Men and women	17,418	536	CHD	Plant-based pattern	Age, sex, race, and age–race interaction, education, household income, and region, total energy intake, smoking and physical activity, BMI, waist circumference, and history of hypertension, dyslipidemia, and diabetes mellitus.
Satija, 2017 [21]	US	NHS	28	30 to 55	Women	73,710	3233	CHD	PBD hPBD uPBD	Age, smoking, physical activity, alcohol intake, multivitamin use, family history of CHD, margarine intake, energy intake, baseline hypertension, hypercholesterolemia, and diabetes, and updated BMI, post-menopausal hormone use (in women)
		NHS2	22	25 to 42	Women	92,329	667			
		HPFS	26	40 to 75	Men	43,259	4731			
Kim, 2019 [7]	US	Atherosclerosis Risk in Communities	25	45 to 64	Men and women	12,168	4381	CVD	PBD hPBD uPBD	Age, sex, race-center, total energy intake, education, smoking status, physical activity, alcohol consumption, and margarine consumption.
Tong, 2019 [10]	UK	EPIC-Oxford study	18.1	≥39	Men and women	16,254	496	IHD	Vegetarians compared with meat eaters	Age, sex, method of recruitment (general practice or postal), and region year of recruitment, education level, Townsend deprivation index, smoking, alcohol consumption, physical activity, dietary supplement use and oral contraceptive and hormone replacement therapy use in women.
							258	Stroke		
Heianza, 2020 [22]	UK	UK Biobank	5	45 to 65	Men and women	156,148	1812	CVD	hPBD	Age, sex, ethnicity, education, parental history of heart disease, smoking habit, physical activity (quintiles), multivitamin use, total energy intake, alcohol consumption, Townsend Deprivation Index, BMI, hypertension, dyslipidemia, and type 2 diabetes
Chiu, 2020 [36]	Taiwan	Tzu Chi Health Study	7	≥49	Men and women	5050	54	Stroke	Vegetarian compared with non-vegetarians	Age, sex, smoking, alcohol drinking, betel nut, leisure time, physical activities, and education plus hypertension, diabetes mellitus, dyslipidemia, ischemic heart disease, and BMI
		Tzu Chi Vegetarian Study	10			8302	121	Stroke		Age, sex, smoking, alcohol drinking, betel nut, leisure time, physical activities, and education plus hypertension, diabetes mellitus, dyslipidemia, ischemic heart disease, and BMI
Shan, 2020 [23]	US	NHS NHS2 HPFS	32	31 to 62	Men and women	209,088	23,366	CVD	hPBD	Age, race/ethnicity, BMI, physical activity, smoking status, alcohol intake, menopausal status, oral contraceptive use, marital status, living alone or with others, family history of myocardial infarction, total energy intake, multivitamin use, and aspirin use.
							18,092	CHD		
							5687	Stroke		
Baden, 2021 [24]	US	NHS NHS2 HPFS	32	25 to 75	Men and women	209,508	6241	Stroke	PBD hPBD uPBD	Age, race, physical activity, alcohol consumption, margarine, total energy intake, smoking, aspirin use, multivitamin use, body mass index, postmenopausal hormone therapy(for women), oral contraceptives, time-varying information on hypertension, hypercholesterolemia, diabetes, antihypertensive use, and anticholesterol medication use.
Glenn, 2021 [25]	US	Women’s Health Initiative	15.3	50 to 79	Women	123,330	13,365	CVD	Plant-based Portfolio Diet score	Age, region, smoking, hormone replacement therapy, dietary modification, calcium and vitamin D, race/ethnicity, education, marital status, hysterectomy history, BMI, physical activity, alcohol intake, energy intake, cancer status, hypertension status, diabetes mellitus status, sodium intake, family history of CVD, family history of diabetes mellitus, hormone therapy use, cholesterol-lowering medication use.
							5640	CHD		
							4400	Stroke		
Petermann-Rocha, 2021 [26]	UK	UK Biobank	8.5	37 to 73	Men and women	7248	1385	CVD	Vegetarians compared with meat eaters	Age, sex, deprivation, ethnicity, comorbidities, smoking, alcohol intake, total sedentary time, physical activity, and BMI.
						7529	65	Stroke		
Chen, 2022 [27]	US	Hispanic Community Health Study/Study of Latinos	6	18 to 74	Men and women	10,293	232	CVD	hPBD	Age, sex, study field center, Hispanic/Latino background, generation status, education, smoking, alcohol consumption, total energy intake, physical activity, BMI, and use of antidiabetic drugs, antihypertensive drugs, or lipid-lowering drugs.
Kouvari, 2022 [28]	Greece	ATTICA study	8.41	>18	Men and women	2020	225	CVD	PBD hPBD uPBD	Age, sex, educational level, family history of CVD, personal history of diabetes mellitus, hypertension and hypercholesterolemia, smoking habits, physical activity level, BMI, alcohol consumption and energy intake
Weston, 2022 [29]	US	Jackson Heart Study	13	21 to 95	Men and women	3635	293	CVD	PBD hPBD uPBD	Age, sex, total energy intake, educational attainment, smoking status, alcohol intake, margarine intake, physical activity, BMI, total cholesterol, hypertension, diabetes, estimated glomerular filtration rate, hormone replacement therapy medication use, and statin medication use.
Gamba, 2023 [30]	Switzerland	CoLaus study	9	35 to 75	Men and women	3721	262	CVD	hPBD	Age, sex, educational level, smoking status, alcohol consumption, physical activity, BMI, total caloric intake, dieting, type 2 diabetes, hypertension, hypercholesterolemia, and family history of CVD.
Li, 2023 [31]	US	Million Veteran Program	8	>18	Men and women	148,506	5025	CVD	PBD hPBD	Age, sex, race/ethnicity, education level, income level, marital status, smoking status, frequency of alcohol consumption, frequency of exercise vigorously, BMI, histories of hypertension, and hypercholesterolemia.
Thompson, 2023 [32]	UK	UK Biobank	10.6 to 12.2	40 to 69	Men and women	123,134	6890	CVD	hPBD uPBD	Age, sex, BMI, race and ethnicity, physical activity level, smoking status, alcohol intake, education level, energy intake, polypharmacy index, multimorbidity index, and aspirin use, stratified by region.
Ganbat, 2024 [33]	Republic of Korea	Health Examiness (Hexa) cohort study	4.2	40 to 79	Men	12,356	2017	CHD	PBD hPBD uPBD	Age, BMI, waist circumference, education level, smoking status, alcohol consumption, household income level, physical activity, energy intake
Park, 2025 [34]	Republic of Korea	Korean Genome and Epidemiology Study (KoGES)_Ansan and Ausung study	18	40 to 69	Men and women	7820	597	CVD	PBD hPBD uPBD	Age, sex, residential area, household income, education level, smoking, alcohol intake, family history of CVD, history of hypertension, BMI, physical activity, and total energy intake
							314	CHD		
							287	Stroke		
Prioux, 2025 [9]	France	NutriNet-Santé study	9	>35	Men and women	63,830	1397	CVD	hPBD uPBD	Age, sex, energy intake, education level, occupation, monthly household income, marital status, number of completed 24 h dietary records, physical activity, diet currently followed for weight management (lose weight or keep it off or stay in shape) or for medical reasons, alcohol consumption, BMI, smoking status, family history of arteritis and/or stroke and/or heart attack and/or high blood pressure and/or high cholesterol and/or diabetes.
						63,831	1051	CHD		
Rui, 2025 [35]	China	PEACE-Shanxi cohort study	2	35 to 75	Men and women	15,090	1242	CVD	PBD	Age, sex, smoking, alcohol consumption, physical activity, marital status, geographical region, education, household income, hypertension, diabetes, dyslipidemia, and BMI

BMI, body mass index; CHD, Coronary Heart Disease; CVD, Cardiovascular disease; EPIC, European Prospective Investigation into Cancer and Nutrition; HPFS, Health Professionals Follow-up Study; hPBD, healthful plant-based diet; IHD, Ischemic heart disease; NHS, Nurses’ Health Study; PBD, plant-based diet; REGARDS, REasons for Geographic and Racial Differences in Stroke; uPBD, unhealthful plant-based diet.

**Table 2:** Summary of pooled relative risks (RR) of cardiovascular disease (CVD) for a plant-based diet (PBD), a healthful plant-based diet (hPBD), and an unhealthful plant-based diet (uPBD).

	No. of Cohorts	RR	95% CI	*p* for Difference
***PBD***				
High *versus* low PBD				
All studies	13	0.90	0.85–0.95	
Stratified by sex				
Male	2	0.94	0.87–1.02	0.38
Female	3	0.90	0.85–0.94	
Stratified by geographical region				
U.S.	9	0.88	0.82–0.94	
Asia	3	0.96	0.87–1.05	0.24 ^a^
Europe	1	0.56	0.14–2.24	0.54 ^a^
Stratified by follow-up years				
≥Median	7	0.90	0.87–0.94	0.45
<Median	6	0.88	0.77–1.00	
***hPBD***				
High *versus* low hPBD				
All studies	13	0.83	0.76–0.90	
Stratified by geographical region				
U.S.	7	0.83	0.74–0.93	
Europe	4	0.75	0.60–0.94	0.59 ^b^
Asia	2	0.91	0.69–1.19	0.63 ^b^
Stratified by follow-up years				
≥Median	8	0.88	0.83–0.92	0.03
<Median	5	0.73	0.63–0.85	
***uPBD***				
High *versus* low uPBD				
All studies	11	1.13	1.03–1.24	
Stratified by sex				
Male	2	1.13	0.98–1.30	0.11
Female	2	1.53	1.31–1.79	
Stratified by geographical region				
U.S.	6	1.10	0.93–1.30	
Europe	3	1.21	1.13–1.29	0.54 ^c^
Asia	2	1.11	0.95–1.30	0.90 ^c^
Stratified by follow-up years				
≥Median	7	1.15	0.99–1.32	0.72
<Median	4	1.10	1.02–1.19	

CI, confidence interval; PBD, plant-based diet; hPBD, healthful plant-based diet; uPBD, unhealthful plant-based diet; RR: relative risk; ^a^ *p* value for difference in RRs of CVD for Asia *versus* US (*p* = 0.24) and Europe *versus* US (*p* = 0.54); ^b^ *p* value for difference in RRs of CVD for Europe *versus* US (*p* = 0.59) and Asia *versus* US (*p* = 0.63); ^c^ *p* value for difference in RRs of CVD for Europe *versus* US (*p* = 0.54) and Asia *versus* US (*p* = 0.90).

## Data Availability

The data presented in this study are available on request from the corresponding author. The data are not publicly available due to privacy.

## Supplementary Materials

The following supporting information can be downloaded at: https://www.mdpi.com/article/10.3390/nu18172779/s1, PRISMA_2020_checklist. Table S1: Search strategy for meta-analysis. Table S2: PICO elements of meta-analysis. Table S3: Quality assessment scores according to the Newcastle-Ottawa scale. References [7,9,10,11,18,19,20,21,22,23,24,25,26,27,28,29,30,31,32,33,34,35,36] are cited in the Supplementary Materials.

## Abbreviations

The following abbreviations are used in this manuscript:

ARIC	Atherosclerosis Risk in Communities
BMI	Body mass index
CAD	Coronary artery disease
CHD	Coronary heart disease
CI	Confidence interval
CVD	Cardiovascular disease
DBP	Diastolic blood pressure
EPIC	European Prospective Investigation into Cancer and Nutrition
GI	Glycemic index
GL	Glycemic load
HDL-C	High-density lipoprotein cholesterol
hPBD	Healthful plant-based diet
HPFS	Health Professionals Follow-up Study
hs-CRP	High-sensitivity C-reactive protein
IHD	Ischemic heart disease
LDL-C	Low-density lipoprotein cholesterol
NHS	Nurses’ Health Study
NPPB	N-terminal pro-BNP
PBD	Plant-based diet
PCI	Percutaneous coronary intervention
PDI	Plant-based dietary index
RCT	Randomized controlled trial
REGARDS	REasons for Geographic and Racial Differences in Stroke
RR	Relative risk
SBP	Systolic blood pressure
TC	Total cholesterol
THBS2	Thrombospondin-2
TG	Triglycerides
TMA	Trimethylamine
TMAO	Trimethylamine N-oxide
uPBD	Unhealthful plant-based diet
