# Supplementary material for: A Plant-Based Diet and Cardiovascular Disease, Coronary Heart Disease, and Stroke: A Systematic Review and Meta-Analysis of Prospective Cohort Studies
Source: Nutrients. 2026 Aug 25;18(17):2779. doi: 10.3390/nu18172779 (PMC13567177; doi:10.3390/nu18172779)
Supplement: Supplementary file 1 [file nutrients-18-02779-s001.zip › nutrients-4408010-supplementary.pdf]

Supplementary Table S1. Search strategy for meta-analysis.

Exposure (#1)	Outcome(#2)	#3
Plant-based diet	Cardiovascular disease	#1 AND #2
Vegetarian	Coronary heart disease	
Vegan	Coronary artery disease	
	Stroke	

Supplementary Table S2. PICO elements of meta-analysis

Population	Adults who have the risk of cardiovascular disease, coronary heart disease, and stroke
Intervention	Plant-based diet
Comparison	High *versus* low adherence to plant-based diet
Outcome	Incidence of cardiovascular disease, coronary heart disease, and stroke

Supplementary Table S3. Quality assessment scores according to the Newcastle-Ottawa scale

Study	Selection (maximum=4)	Comparability (maximum=2)	Outcome (maximum=3)	Total score (maximum=9)
Crowe, 2013 [18]	3	2	3	8
Judd, 2013 [19]	3	2	3	8
Shikany, 2015 [20]	3	2	3	8
Satija, 2017 [21]	2	2	3	7
Kim, 2019 [7]	4	2	3	9
Tong, 2019 [10]	3	2	3	8
Heianza, 2020 [22]	3	2	3	8
Chiu, 2020 [36]	2	2	3	7
Shan, 2020 [23]	2	2	3	7
Baden, 2021 [24]	2	2	3	7
Glenn, 2021 [25]	3	2	3	8
Petermann-Rocha, 2021 [26]	3	2	3	8
Chen, 2022 [27]	4	2	2	8
Kouvari, 2022 [28]	3	2	3	8
Weston, 2022 [29]	3	2	3	8
Gamba, 2023 [30]	3	2	3	8
Li, 2023 [31]	3	2	3	8
Thompson, 2023 [32]	3	2	3	8
Ganbat, 2024 [33]	3	2	2	7
Park, 2025 [34]	3	2	3	8
Prioux, 2025 [9]	3	2	3	8
Rui, 2025 [35]	3	2	1	6

References [7,9,10,18-36] are cited in the table.
